# Identification of the domains of the influenza A virus M1 matrix protein required for NP binding, oligomerization and incorporation into virions

**DOI:** 10.1099/vir.0.82809-0

**Published:** 2007-08

**Authors:** Sarah L. Noton, Elizabeth Medcalf, Dawn Fisher, Anne E. Mullin, Debra Elton, Paul Digard

**Affiliations:** Division of Virology, Department of Pathology, University of Cambridge, Tennis Court Road, Cambridge CB2 1QP, UK

## Abstract

The matrix (M1) protein of influenza A virus is a multifunctional protein that plays essential structural and functional roles in the virus life cycle. It drives virus budding and is the major protein component of the virion, where it forms an intermediate layer between the viral envelope and integral membrane proteins and the genomic ribonucleoproteins (RNPs). It also helps to control the intracellular trafficking of RNPs. These roles are mediated primarily via protein–protein interactions with viral and possibly cellular proteins. Here, the regions of M1 involved in binding the viral RNPs and in mediating homo-oligomerization are identified. *In vitro*, by using recombinant proteins, it was found that the middle domain of M1 was responsible for binding NP and that this interaction did not require RNA. Similarly, only M1 polypeptides containing the middle domain were able to bind to RNP–M1 complexes isolated from purified virus. When M1 self-association was examined, all three domains of the protein participated in homo-oligomerization although, again, the middle domain was dominant and self-associated efficiently in the absence of the N- and C-terminal domains. However, when the individual fragments of M1 were tagged with green fluorescent protein and expressed in virus-infected cells, microscopy of filamentous particles showed that only full-length M1 was incorporated into budding virions. It is concluded that the middle domain of M1 is primarily responsible for binding NP and self-association, but that additional interactions are required for efficient incorporation of M1 into virus particles.

## INTRODUCTION

The influenza A virus matrix protein M1 is a multifunctional protein playing many essential roles throughout the virus life cycle. M1 forms the major structural component of the virion, lying beneath a lipid envelope containing the viral haemagglutinin (HA) and neuraminidase (NA) glycoproteins and the M2 ion channel (Nayak *et al.*, 2004). M1 in turn surrounds the genomic ribonucleoproteins (RNPs). RNPs consist of the viral RNA polymerase and a chain of nucleoprotein (NP) monomers, around which the negative-sense RNA segments are wrapped (Portela & Digard, 2002). M1 is also complexed with small quantities of the viral nuclear export protein (NEP/NS2) in the virion (Yasuda *et al.*, 1993). As well as being the most abundant polypeptide in virions, M1 drives virus budding. Expression of M1 alone in cells produces virus-like particles (Gomez-Puertas *et al.*, 2000; Latham & Galarza, 2001), whilst in the context of authentic virus, M1 amino acid sequence polymorphisms control particle shape (Bourmakina & Garcia-Sastre, 2003; Elleman & Barclay, 2004). Vesicular budding of M1 in the absence of other viral proteins reflects its ability to bind lipid membranes (Gregoriades, 1980), although, in infected cells, interactions with the cytoplasmic tails of the viral membrane proteins may also be important (Enami & Enami, 1996; Ali *et al.*, 2000; Zhang *et al.*, 2000). Budding presumably also depends on the ability of M1 to oligomerize (Sha & Luo, 1997; Zhao *et al.*, 1998; Ruigrok *et al.*, 2000).

M1 also controls the intracellular trafficking of RNPs. During entry of the virus, the M1–RNP interaction must be disrupted to enable transport of RNPs into the nucleus (Martin & Helenius, 1991b; Bui *et al.*, 1996). M1 also regulates RNP nuclear export (Martin & Helenius, 1991a; Bui *et al.*, 2000). Following the ‘late’ synthesis of M1, some enters the nucleus (Bucher *et al.*, 1989) and interacts with RNPs. Following this, NEP binds to M1 to form a ‘daisy chain’ of proteins (Yasuda *et al.*, 1993; Akarsu *et al.*, 2003). NEP links this complex with the cellular nuclear-export protein CRM1 (O'Neill *et al.*, 1998; Neumann *et al.*, 2000; Elton *et al.*, 2001), which mediates RNP export.

**Fig. 1. f1:**
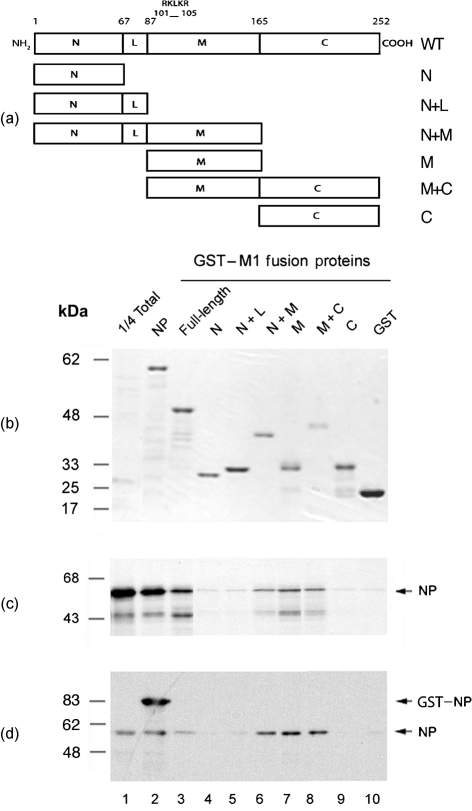
Domain structure and NP-binding activity of M1. (a) Schematic diagram of the domain structure of M1 as defined by crystallography (coordinates are amino acid number) and the subdivisions used in this study. Also shown is the location of the RKLKR motif important in binding RNA and NEP. (b–d) NP-binding activity of GST, the indicated GST–M1 fusion proteins or GST–NP (NP). [^35^S]Methionine-radiolabelled, *in vitro*-translated NP (b, c) or purified NP (d) was incubated with 6 μg of each fusion protein attached to glutathione–Sepharose beads and bound material was analysed by SDS-PAGE and (b) Coomassie blue staining (to visualize the input GST-fusion proteins), (c) autoradiography or (d) Western blot analysis with anti-NP. Aliquots equivalent to one-quarter of the input soluble NP (1/4 Total) were also analysed to provide a guide to binding efficiency. Molecular mass markers are indicated on the left and the position of specified proteins on the right. The approximately 43 kDa radiolabelled species visible in (c) is an aberrant NP *in vitro* translation product.

The M1 polypeptide possesses N-terminal (N), linker (L), middle (M) and C-terminal (C) domains (Fig. 1a[Fig f1]). The N, L and M sequences have been analysed by X-ray diffraction (Sha & Luo, 1997; Harris *et al.*, 2001; Arzt *et al.*, 2004). These studies show that the N and M domains are *α*-helical bundles linked by a short helix (L domain). Circular dichroism spectroscopy suggests that the C-terminal domain also has an *α*-helical structure (Arzt *et al.*, 2001). The M and C domains are separated by a zinc finger-like motif that is thought to act as an interdomain linker (Wakefield & Brownlee, 1989; Elster *et al.*, 1994; Arzt *et al.*, 2001).

Thus, M1 plays key roles in controlling RNP trafficking and virion assembly through a web of protein–protein interactions. Currently, there is controversy over which domain(s) of M1 interacts with RNPs. Ye *et al.* (1999) concluded that the N-terminal domain of M1 mediates a protein–protein contact with NP, whilst a basic RNA-binding motif, ^101^RKLKR^105^ (Wakefield & Brownlee, 1989; Watanabe *et al.*, 1996; Elster *et al.*, 1997), located in the middle domain, interacts with the viral RNA (vRNA). In diametric contrast, Baudin *et al.* (2001) found that the C-terminal domain of M1 bound to RNPs or NP alone, but that the N+M domains did not. Due to these unresolved discrepancies, mutational studies of M1 using reverse genetics to test hypotheses of how the protein functions cannot be interpreted fully.

The aim of this paper was to identify the domains of M1 that are necessary for interacting with RNPs and/or NP, for oligomerization and for incorporation into virus particles. The middle domain of M1 was found to play an important role in both oligomerization and RNP–NP interactions. However, only full-length M1 was incorporated into budding viral particles, suggesting that additional interactions other than self-association and RNP binding are necessary for virion assembly.

## METHODS

### Cells and viruses.

Madin–Darby canine kidney (MDCK) cells were cultured as described previously (Carrasco *et al.*, 2004). A filamentous variant (PR8/MUd) of virus strain A/PR/8/34 (PR8) was created by reverse genetics using the PR8 clones described by de Wit *et al.* (2004), except for segment 7, which was from A/Udorn/72 (Elleman & Barclay, 2004). For biochemical analyses, egg-grown virus (a vaccine strain reassortant between PR8 and A/Johannesburg/33/94) was gradient-purified as described previously (Blok *et al.*, 1996).

### Plasmids.

Plasmids expressing PR8 NP fused to glutathionine *S*-transferase (GST) or maltose-binding protein (MBP) have been described previously (Digard *et al.*, 1999). Plasmid pGFPM703 that expresses full-length PR8 M1 fused to green fluorescent protein (GFP) was described by Simpson-Holley *et al.* (2002). To construct plasmids expressing the various domains of M1, regions of the gene were PCR-amplified from a cDNA clone of PR8 M1 (Young *et al.*, 1983) and cloned into either pGEX-3X (Pharmacia; for expression as GST-fusion proteins), pEGFP-c2 (Clontech; for expression as GFP-fusion proteins) or pKT-0 (Blok *et al.*, 1996; for *in vitro* expression of untagged protein from a T7 RNA polymerase promoter). PCR primers were designed by using the domain boundaries assigned by Sha & Luo (1997) (Fig. 1a[Fig f1]). Forward primers included a common *Bgl*II restriction site and an ATG codon (5′-CTCAGATCTCGATG), whilst reverse primers included a downstream sequence (5′-CGAATTCTCA) with an *Eco*RI site for subcloning purposes and a stop codon. The unique sequences used were 5′-AGTCTTCTAACC (forward primer to amplify from codon 1 onwards), 5′-GGGGATCCAAATAAC (to amplify from codon 88 onwards), 5′-GTGACAACAACC (to amplify from codon 165 onwards), 5′-GAGCGTGAACACAAA (reverse primer to amplify backwards from codon 67), 5′-GTTCCCATTAAGGGC (to amplify backwards from codon 88), 5′-TTGCCTATGAGACCG (to amplify backwards from codon 165) and 5′-CTTGAACCGTTG (to amplify backwards from codon 252). Pairs of forward and reverse primers were used to amplify full-length M1, N, N+L, N+M, M, M+C and C domain sequences to clone into pGEX-3X, and full-length M1, N+M, M, M+C and C domain sequences to clone into pEGFP-c2. N and N+L domain GFP and pKT constructs were made by digesting pGFPM703 with *Pst*I or *Bam*HI to truncate the M1 ORF at codons 75 and 90, respectively.

### Antibodies.

Antisera against NP (2915) were raised by immunizing rabbits with MBP–NP. Antisera against PR8 virus were described previously (Amorim *et al.*, 2006). Anti-GFP antibody JL8 was obtained from Clontech. Horseradish peroxidase-conjugated antibodies for Western blot analysis were obtained from GE Healthcare. For immunofluorescence microscopy, anti-rabbit IgG conjugated to Alexa 594 (Molecular Probes) was used.

### Protein expression and purification.

GST-tagged M1, NP and MBP–NP fusion proteins were expressed in *Escherichia coli* TG1 cells and purified by affinity chromatography on glutathione–Sepharose (GE Healthcare) or amylose resin (New England Biolabs), respectively (Digard *et al.*, 1999). A salt wash (1 M NaCl for GST- and 2 M NaCl for MBP-fusion constructs) was included to remove co-purifying bacterial RNA (Wakefield & Brownlee, 1989; Digard *et al.*, 1999). Purified NP was obtained by removal of the MBP moiety from the MBP–NP fusion protein (Elton *et al.*, 1999b). NP and M1 proteins were radiolabelled with [^35^S]methionine in rabbit reticulocyte lysate (Promega) by using a coupled *in vitro* transcription–translation system (Craig *et al.*, 1992).

### Protein-binding assays.

One microlitre of *in vitro*-translated protein or 0.3 μg purified NP protein was mixed with 100 μl IP buffer [100 mM KCl, 50 mM Tris/Cl (pH 7.6), 5 mM MgCl_2_, 1 mM dithiothreitol (DTT), 0.1 % Nonidet P-40] and incubated with 6 μg (unless otherwise stated) GST-fusion protein attached to 40 μl glutathione–Sepharose beads. The reaction was incubated for 1 h at room temperature and then centrifuged to collect the solid phase. The pellet was washed three times with 750 μl IP buffer and bound proteins were eluted by boiling in SDS-PAGE sample buffer. Samples were separated by SDS-PAGE and analysed by staining with Coomassie brilliant blue dye and autoradiography, or by Western blot (Elton *et al.*, 1999a).

### RNP co-sedimentation assay.

Purified virus (approx. 15 μg) was lysed by dilution into 17.5 μl band-shift buffer [20 mM Tris/Cl (pH 7.6), 50 mM NaCl, 5 mM MgCl_2_, 0.5 mM DTT] containing 0.5 % Nonidet P-40 and incubated with 2.5 μl *in vitro* translation mixture. The reactions were layered on top of 100 μl 20 % sucrose in band-shift buffer and centrifuged at 120 000 ***g***_av_ for 15 min at 4 °C in a Beckman benchtop ultracentrifuge using a TLA 100 rotor, to separate viral lipid and other low-density material from virion cores containing M1 and RNPs. Pellet and supernatant fractions were analysed by SDS-PAGE, Coomassie staining and autoradiography.

### Transfection and infection of cells.

MDCK cells (4×10^5^ per well) were transfected in suspension with 0.8 μg plasmid by using Lipofectamine (Invitrogen) according to the manufacturer's instructions and seeded into 24-well plates. After 24 h, cells were superinfected with PR8/MUd virus at an m.o.i. of 5. Twelve hours later, cells were fixed in PBS containing 4 % formaldehyde and stained for surface HA and NA with anti-PR8 serum as described previously (Simpson-Holley *et al.*, 2002). Fluorescent emissions were imaged by using a Leica TCS-NT confocal microscope (Simpson-Holley *et al.*, 2002).

## RESULTS

### Identification of M1 domains involved in NP binding

Our aim was to map the M1 amino acid sequences responsible for NP binding through deletion mutagenesis of M1. Many earlier studies mapping the interaction of M1 with RNPs employed the strategy of using either convenient restriction-enzyme sites to construct gene deletions (Watanabe *et al.*, 1996; Ye *et al.*, 1999) or chemical treatment of the protein (Ye *et al.*, 1987, 1989) to create M1 fragments. However, with the currently available high-resolution structural information, it was possible to design a set of M1 deletion mutants that corresponded to the domain structure of the polypeptide as revealed by X-ray crystallography, circular dichroism spectroscopy and structure-prediction algorithms (Sha & Luo, 1997; Arzt *et al.*, 2001; Harris *et al.*, 2001). Accordingly, a set of mutants corresponding to the N, L, M and C domains were created as gene fusions with the C terminus of GST (Fig. 1a[Fig f1]). A plasmid encoding full-length M1 (WT) fused to GST was also created. All seven fusion proteins were successfully expressed and purified from *E. coli* as reasonably homogeneous preparations (Fig. 1b[Fig f1], lanes 3–9).

**Fig. 2. f2:**
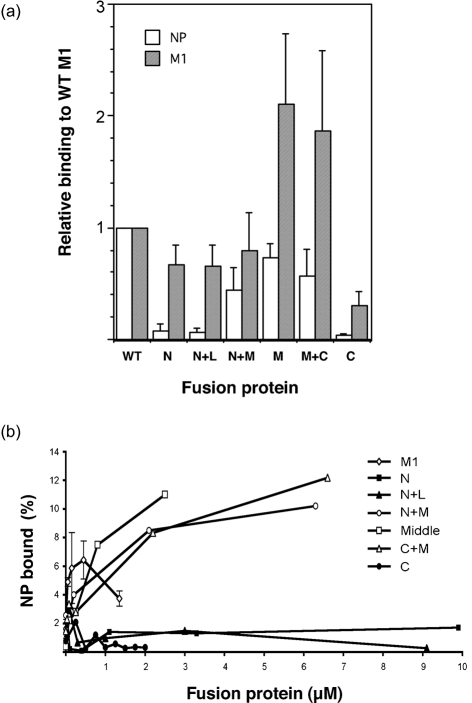
Quantitative analysis of NP- and M1-binding activity of the GST–M1 fusion proteins. (a) The amount of bound radiolabelled NP or M1 from three independent assays with the indicated GST-fusion proteins was quantified by densitometry. Values were corrected by the subtraction of any background seen with GST only, normalized with respect to the amount bound by WT M1, and plotted as the mean±sd. (b) The amount of radiolabelled NP bound by increasing concentrations of the indicated GST-fusion proteins was quantified similarly and expressed as the percentage of input material bound. Values are from a single representative experiment, except for full-length GST–M1, where the mean±range of three experiments is plotted.

PR8 NP was radiolabelled with [^35^S]methionine by *in vitro* transcription–translation and tested for its ability to bind the GST–M1 fusion proteins, GST alone (as a negative control) or GST–NP (as a positive control) in pull-down assays. From the Coomassie-stained gel (Fig. 1b[Fig f1]), it could be seen that approximately equal amounts of the fusion proteins were added to each binding reaction. Autoradiography revealed that, as expected (Elton *et al.*, 1999a), only trace amounts of NP bound to GST alone (Fig. 1c[Fig f1], lane 10), but large quantities bound to GST–NP (lane 2). In comparison to GST–NP, full-length GST–M1 bound less NP, but still well above background levels (compare lanes 3 and 10). The M1 N, N+L or C domains displayed only background amounts of NP binding (lanes 4, 5 and 9). In contrast, fusion proteins containing the middle domain (N+M, M and M+C) displayed NP-binding activity similar to that of the full-length GST–M1 fusion protein (lanes 6–8). Replicate experiments were performed and quantified by densitometry. Results confirmed that the N, N+L and C domains bound amounts of NP that were only slightly above background (Fig. 2a[Fig f2]). In contrast, any fusion protein containing the M domain possessed substantial binding ability, with the M domain alone approaching that observed for the full-length protein (Fig. 2a[Fig f2]).

The interaction between M1 and NP was characterized further by titrating a constant amount of NP with a range of GST fusion-protein concentrations. As before, the N, N+L and C domains bound only small amounts of NP, even with increasing concentrations of fusion protein (Fig. 2b[Fig f2]). However, fusion proteins containing the M domain displayed much higher levels of NP-binding activity that titrated with increasing concentrations of ligand before reaching a plateau. Full-length M1–GST also exhibited titratable NP binding but, unlike the separate domains, NP binding at higher concentrations decreased reproducibly, rather than reaching a constant maximum.

To confirm further that the middle domain of M1 interacts with NP, the protein-binding assay was repeated ‘in reverse’. Untagged WT radiolabelled M1, the M1 deletion mutants and NP were *in vitro*-translated in rabbit reticulocyte lysate and tested for their ability to bind either GST–NP or GST alone.

**Fig. 3. f3:**
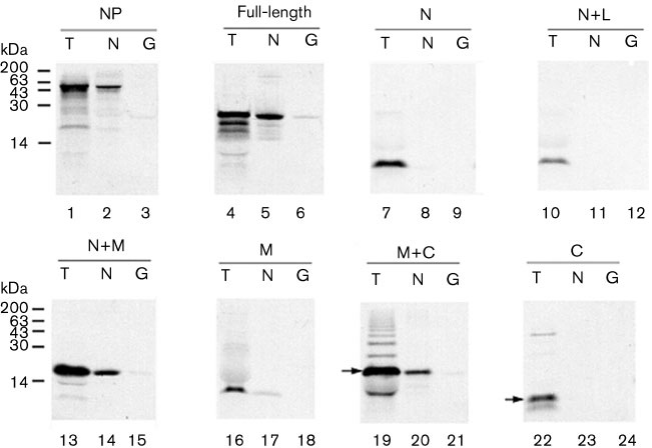
Ability of *in vitro*-translated M1-domain fragments to bind to GST–NP. Aliquots of the indicated radiolabelled polypeptides were analysed by SDS-PAGE and autoradiography before (T) or after binding to GST–NP (N) or GST (G). Molecular mass markers are indicated on the left; arrows indicate the expected protein sizes for M+C and C sub-fragments.

Only trace amounts of radiolabelled NP bound to GST (Fig. 3, lane 3) and strong self-association was observed (lane 2). Full-length M1, N+M, M and M+C domains also bound to GST–NP (lanes 5, 14, 17 and 20) and exhibited only background binding to GST (lanes 6, 15, 18 and 21). No detectable binding to NP was seen with the N, N+L or C domains (lanes 7–12, 22–24). These results support the finding that the middle domain of M1 mediates binding to NP.

### Role of RNP organization in M1 binding

Previous studies examining M1–RNP interactions that utilized authentic RNPs as the binding target suggested that M1 binds via both M1–NP and M1–RNA interactions (Elster *et al.*, 1997; Perez & Donis, 1998; Ye *et al.*, 1999). The experimental system that we used above does not contain genuine RNPs, because neither vRNA nor the viral polymerase are present. However, the *in vitro* transcription–translation system generates large quantities of single-stranded RNA and, because NP binds RNA without apparent sequence specificity (Portela & Digard, 2002), it is possible that NP–RNA complexes were formed that might behave similarly to RNPs. It is also relevant that the M domain of M1 contains an RNA-binding motif (Ye *et al.*, 1989; Watanabe *et al.*, 1996; Elster *et al.*, 1997). Accordingly, to investigate further whether NP–RNA complexes were involved in M1 interactions in our system, we used purified NP protein in which salt washes during affinity purification, followed by heparin–agarose chromatography, ensured that the protein was free of RNA (Elton *et al.*, 1999b). Purified NP bound well to GST–NP, but only background amounts bound to GST (Fig. 1d[Fig f1]). The GST-fusion proteins containing the N, N+L and C domains of M1 displayed poor affinity for the purified NP but, again, strong binding was observed with N+M, M and M+C fusion proteins. Overall, there was no significant difference in behaviour between binding assays using radiolabelled NP in a complex cell extract and those using purified NP with regards to the relative binding activities of the M1 domains. Furthermore, identical results were obtained with an NP RNA-binding mutant (S314N; Medcalf *et al.*, 1999), and RNase treatment of the rabbit reticulocyte lysates did not alter the pattern of binding specificities (data not shown). Thus, both binding assays examine predominantly M1–NP interactions and these are mediated primarily by the M domain of M1.

**Fig. 4. f4:**
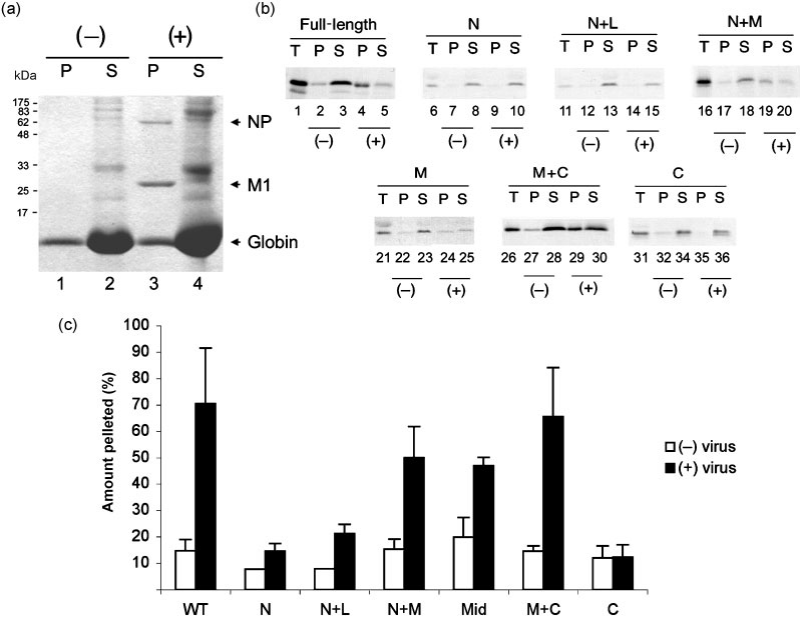
Virion RNP core co-sedimentation assay. Aliquots of the indicated radiolabelled, *in vitro*-translated M1 polypeptides were incubated in the presence (+) or absence (−) of detergent-disrupted virus and analysed by SDS-PAGE and (a) Coomassie blue staining or (b) autoradiography before (T) or after separation into pellet (P) and supernatant (S) fractions by centrifugation. (a) Molecular mass markers are indicated on the left and the position of specified proteins on the right. (c) The percentage of M1 polypeptides in the pellet fraction was quantified by densitometry. The mean±range of two or three independent experiments is plotted.

Interactions between M1 and non-RNP NP are not necessarily the same as interactions between M1 and RNPs. Accordingly, we tested binding of radiolabelled M1 fragments to authentic RNPs. RNPs were obtained by lysing purified virus with non-ionic detergent and then incubated with *in vitro*-translated WT and deletion-mutant M1 polypeptides. After centrifugation through a 20 % sucrose cushion to separate virion cores (comprising RNPs and associated M1) from lipid and other low-density material, the pellet and supernatant fractions were collected and analysed by SDS-PAGE. Coomassie blue staining revealed rabbit globin and ribosomal and viral envelope proteins to be in the supernatant fraction, whereas NP and M1 were in the pellet, representing virion cores (Fig. 4a[Fig f4]). Autoradiograms showed that exogenous, full-length M1 partitioned mainly to the supernatant in the absence of lysed virus, whereas in the presence of purified virus, most of the radiolabelled M1 was found in the pellet, indicating that it interacted with the virion cores (Fig. 4b[Fig f4], lanes 1–5). A similar pattern was also observed with the N+M deletion mutant (lanes 16–20). The middle domain of M1 and the M+C fragment also displayed substantial levels of binding to virion cores (lanes 21–30), but the majority of the N, N+L and C fragments remained in the supernatant, even in the presence of virion cores (lanes 6–15, 31–36). Quantification of replicate experiments confirmed that substantial amounts of M1 polypeptides containing the middle domain co-pelleted with RNPs, whilst the N and N+L domains bound poorly and the C-terminal domain at background levels (Fig. 4c[Fig f4]). Thus, consistent with previous assays, the middle domain of M1 mediates binding to authentic virion cores.

### M1 oligomerization

**Fig. 5. f5:**
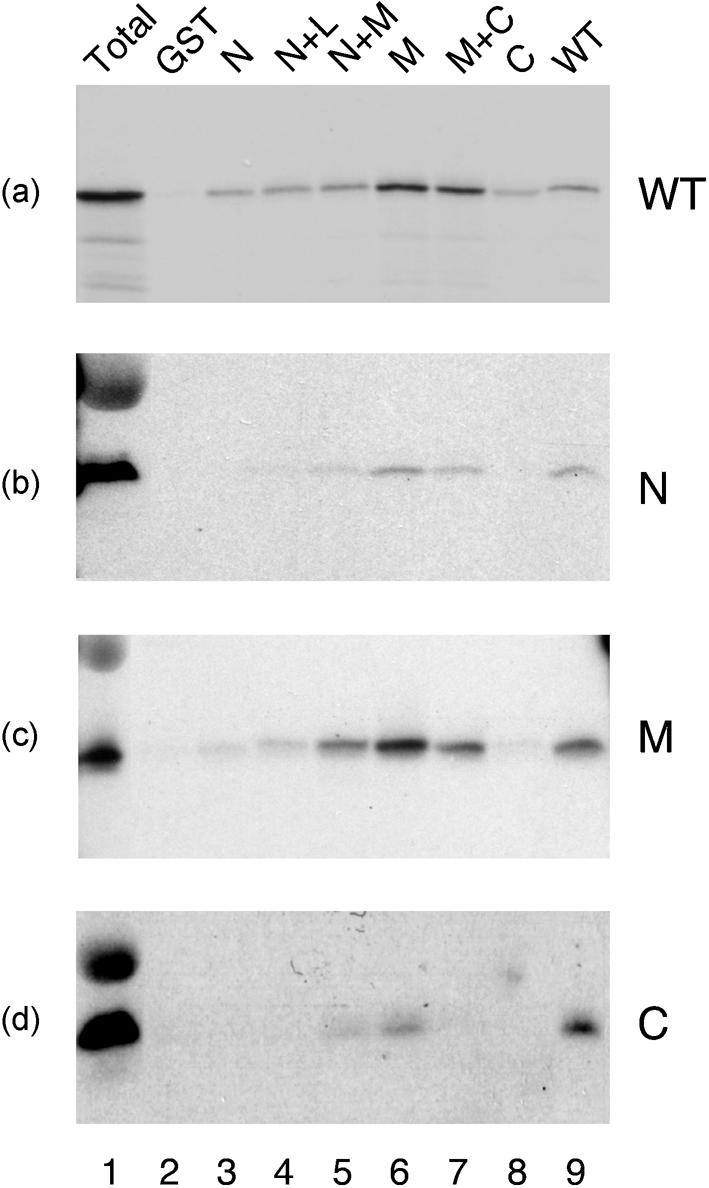
Self-association of M1. Aliquots of the indicated radiolabelled, *in vitro*-translated M1 polypeptides were analysed by SDS-PAGE and autoradiography before (T) or after binding to a panel of GST–M1 fusion proteins.

During viral assembly, M1 is thought to drive budding at the cell surface through its ability to interact with the plasma membrane and to oligomerize (Sha & Luo, 1997; Zhao *et al.*, 1998; Gomez-Puertas *et al.*, 2000; Ruigrok *et al.*, 2000). Indeed, the virion core-binding assay (Fig. 4) could potentially reflect M1–M1 interactions as well as RNP binding. Accordingly, to gain a better understanding of the mechanism of M1–M1 polymerization, the GST–M1 fusion constructs were tested for their ability to interact with *in vitro*-translated M1 polypeptides. WT radiolabelled M1 bound to full-length GST–M1, but not to GST alone (Fig. 5a[Fig f5], lanes 2 and 9), indicating self-association in the absence of a membrane surface. Self-association of full-length M1 was also seen with all the GST–M1 sub-fragments (Fig. 5a[Fig f5], lanes 3–8). In replicate experiments, the N, N+L and N+M domain constructs bound similar amounts of *in vitro*-translated M1 to the full-length GST–M1 fusion protein, whereas the C-terminal domain had around 40 % of WT-binding activity (Fig. 2a[Fig f2]). For the N, N+L and C fragments, this contrasts with their almost total lack of NP-binding activity (Fig. 2a[Fig f2]). The M and M+C proteins showed the strongest affinity for WT M1 (Fig. 5a[Fig f5], lanes 6 and 7), with on average twice the binding activity of the full-length GST-fusion protein (Fig. 2a[Fig f2]). Crystal structures of the N+M domains suggest that they dimerize through M–M domain contacts at both neutral and acidic pH, with additional N–N domain interactions at neutral pH (Sha & Luo, 1997; Arzt *et al.*, 2001; Harris *et al.*, 2001). To test whether these interactions occur in solution, we next examined binding of individual M1 domains to the panel of GST–M1 fusion proteins. In confirmation of the intersubunit interactions seen in both neutral- and acidic-pH structures, the M domain self-associated strongly (Fig. 5c[Fig f5], lane 6). It also bound well to the N+M, M+C and full-length GST-fusion proteins, but weakly to the N and N+L and C domain constructs (Fig. 5c[Fig f5]). The isolated N domain did not interact strongly with any of the GST–M1 fusion constructs, but bound best to the full-length protein and to the M and M+C peptides (Fig. 5b[Fig f5]). No detectable self-interactions were seen between N domains, and only very weak binding occurred to the N+L protein (Fig. 5b[Fig f5], lanes 3 and 4). Similar patterns of reactivity were seen when *in vitro*-translated N+L polypeptide was used as the target (data not shown). The C domain bound reasonably well to full-length GST–M1 (Fig. 5d[Fig f5], lane 9) and weakly to the M and N+M ligands (lanes 5 and 6). Overall, we conclude that the M domain of M1 is the main determinant of self-association, but that the N- and C-terminal domains make significant contributions.

**Fig. 6. f6:**
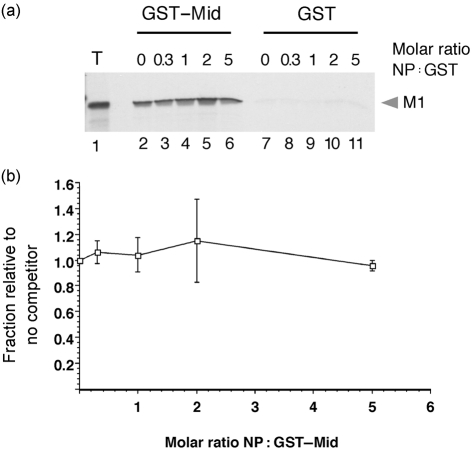
Effect of excess NP on M1 self-association. (a) Aliquots of radiolabelled, *in vitro*-translated WT M1 were analysed by SDS-PAGE and autoradiography before (T) or after binding to GST–middle domain (GST–Mid) or GST in the presence of the indicated amounts of NP. (b) Bound M1 was quantified by densitometry and plotted (mean±range of two independent experiments) relative to the amount bound in the absence of NP.

As the same domain of M1 plays roles in binding NP and oligomerization, the question arises as to whether the interactions are competitive. To test this, we examined the ability of the M1 middle domain to bind full-length radiolabelled M1 in the presence of increasing amounts of purified NP. As before, WT M1 bound strongly to the immobilized GST–middle domain fusion protein, but not to GST alone (Fig. 6a[Fig f6], lanes 2 and 7). However, this was not altered significantly by the addition of up to a fivefold molar excess (with respect to the GST polypeptides) of NP (lanes 3–6, 8–11). When replicate experiments were quantified, M1 self-association was seen to be independent of NP concentration (Fig. 6b[Fig f6]). Thus, NP does not interfere with M1 self-association.

### Domain requirements for incorporation of M1 into virions

**Fig. 7. f7:**
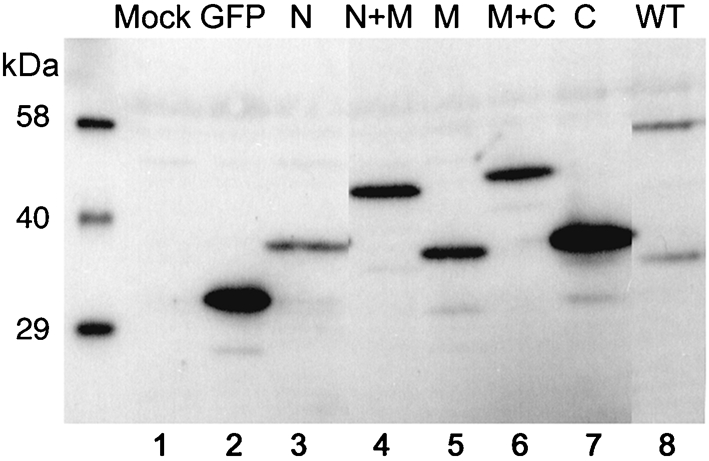
Expression of GFP–M1 fusion proteins in MDCK cells. Lysates from cells transfected (or mock-transfected) with plasmids expressing the indicated GFP-fusion proteins were separated by SDS-PAGE and analysed by Western blot with anti-GFP serum. Molecular mass markers are indicated on the left.

**Fig. 8. f8:**
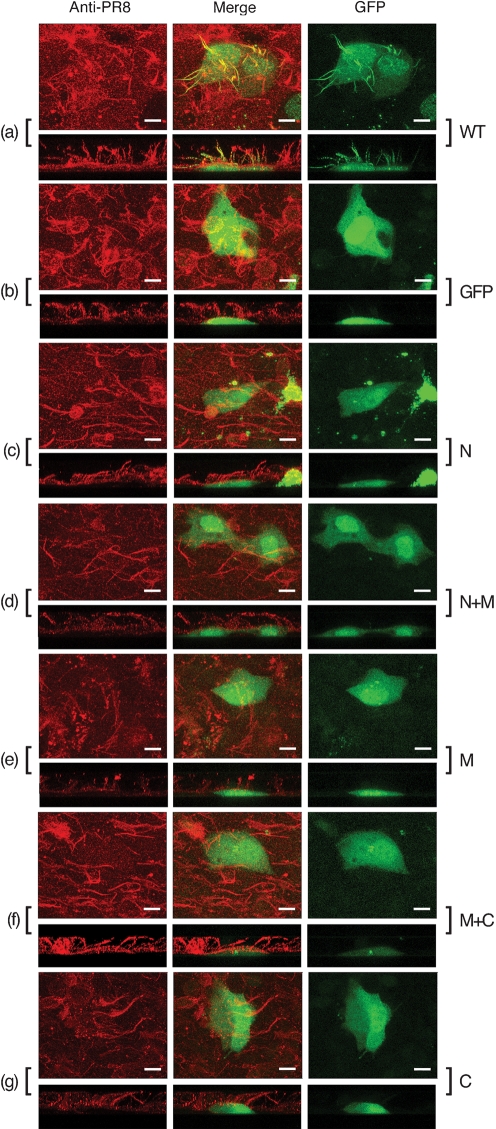
Incorporation of GFP–M1 polypeptides into filamentous viral particles. Cells were transfected with plasmids expressing the indicated GFP-fusion proteins and superinfected with PR8/MUd virus. At 12 h post-infection, cells were fixed and the cell surface was stained with anti-PR8 serum (in red) and imaged for red and green fluorescence. The images shown are projections (made using Leica TCS-SP software) of merged confocal stacks taken across the cells at approximately 0.5 μm intervals in the *xy* plane (upper panels) and approximately 0.8 μm intervals in the *xz* plane (lower panels). Post-capture processing to allow daylight visualization of the images was performed by using Adobe Photoshop. Bars, 10 μm.

Based on the observation that the middle domain of M1 mediates an interaction with both RNPs and itself, we tested whether this was sufficient for M1 incorporation into budding viral particles. For this, we took advantage of the fact that certain strains of influenza A virus produce micrometre-length filamentous particles and of our previous demonstration that incorporation of full-length M1 fused to GFP into these virions is visualized readily by fluorescence microscopy (Simpson-Holley *et al.*, 2002). Accordingly, a set of plasmids encoding the various domains of M1 fused to the C terminus of GFP were transfected into MDCK cells. Following overnight incubation, Western blot analysis of cell lysates using anti-GFP serum confirmed that all five sub-fragments of M1, along with GFP itself and the WT GFP–M1 fusion protein, expressed polypeptides of the expected size (Fig. 7). Another set of transfected cells was superinfected with the filamentous PR8/MUd virus. At 12 h post-infection, cells were fixed and the cell surfaces were stained for viral glycoproteins and analysed by confocal microscopy. This revealed the presence of profuse filamentous structures on the surface of the infected cells (Fig. 8), but not mock-infected cells (data not shown). When infected cells were imaged in the *z*-axis, the filaments were visualized clearly as structures exceeding 10 μm in length projecting from the apical surfaces of the cells (Fig. 8, lower panels). In cells expressing WT M1 fused to GFP, obvious green, filamentous structures were formed that co-localized with the anti-PR8 staining (Fig. 8a[Fig f8]), indicating efficient packaging of this fusion protein into virus particles. As observed previously (Simpson-Holley *et al.*, 2002), similar packaging of GFP alone was not seen (Fig. 8b[Fig f8]). However, no significant incorporation of any of the M1 deletion mutants was observed, despite a proportion of each protein being resident in both the nucleus and cytoplasm and thus at least potentially available for interactions with RNPs and/or the plasma membrane (Fig. 8c–g[Fig f8]). Therefore, although the M domain of M1 mediates efficient binding to NP and self-association, it is not sufficient for incorporation into virus particles, suggesting that additional interactions are necessary.

## DISCUSSION

Previous studies mapping the regions of M1 responsible for binding RNPs have given contradictory results. Ye *et al.* (1999) concluded that the association involves aa 1–135 of M1, encompassing the N-terminal domain, and helices 6–8 of the middle domain, and found no activity from C-terminal fragments of M1. In contrast, Baudin *et al.* (2001) concluded the C-terminal domain of M1 mediated the RNP–NP association, but saw no activity from the N+M domain. The involvement of vRNA in the M1–RNP interaction has also proved contentious, with one study finding it essential (Melnikov *et al.*, 1985), another (Ye *et al.*, 1999) a contributory factor, whereas Baudin *et al.* (2001) thought it irrelevant. We agree that RNA is not required for an M1–NP interaction, but cannot rule it out as a contributory factor. Regarding the domains of M1 involved in binding RNPs, our results are in broad agreement with the findings of Ye *et al.* (1999), in that we find activity from the N+M domains, but conflict with those of Baudin *et al.* (2001). Our results are also consistent with reverse-genetics studies showing that mutation of arginine residues in the basic stretch of the middle domain weakened M1–RNP interactions and reduced virus viability (Liu & Ye, 2004). The reasons for the discrepancies regarding the NP-binding activity of the M1 C-terminal domain are not clear. Ye *et al.* (1999) studied A/WSN/33 virus, whereas we and Baudin *et al.* (2001) used PR8, so strain-specific differences seem an unlikely explanation. Ye *et al.* (1999) expressed M1 fragments in rabbit reticulocyte lysate, whereas Baudin *et al.* (2001) used *E. coli*. Here, we used both approaches with identical results, so the choice of expression systems does not explain the discrepancies. We have not examined the folding of the proteins used here directly, but their ready expression in a variety of systems and evident activity in oligomerization are not consistent with global misfolding.

Crystallographic packing of N+M domain monomers suggests the possibility that M1 oligomerization occurs via homopolymeric interactions between the M and N domains (Sha & Luo, 1997; Arzt *et al.*, 2001; Harris *et al.*, 2001). Consistent with this, we found that M1 self-association in solution was driven primarily by the M domain, with a weaker contribution from the N-terminal domain. Further supporting the importance of the M domain in M1 oligomerization, Baudin *et al.* (2001) found that mutations in helix 6 resulted in reduced polymerization of the protein. Crystallographic analyses have yet to provide information on the disposition of the C-terminal domain, and a model for M1 oligomerization in virions proposed that it lies out of the plane of the N+M domain ribbon towards the interior of the particle, making little contribution to the lattice (Harris *et al.*, 2001). However, our data suggest that the C-terminal domain does participate in M1–M1 interactions via the M domain. This is perhaps consistent with the results of tritium-bombardment experiments indicating that the C-terminal domain is not buried in the interior of the virus particle (Shishkov *et al.*, 1999). If one accepts the plausible hypothesis that M1 amino acid sequence polymorphisms control virion shape through subtle differences in packing, then our results are also consistent with experiments mapping the filamentous virion phenotype to sequences in the N, M and C domains (Bourmakina & Garcia-Sastre, 2003; Elleman & Barclay, 2004; Burleigh *et al.*, 2005).

Although the middle domain of M1 was sufficient to bind NP and M1 itself, only the full-length protein was recruited into filamentous virions, raising the possibility that the N- and C-terminal domains of M1 are important for interaction(s) with other cellular and/or viral substrates necessary for incorporation into virions. We hypothesize that, in the absence of this/these interaction(s), fragments of M1 containing the middle domain that are able to self-associate and bind NP are nevertheless outcompeted by authentic M1 for assembly into the budding virion. Candidate viral factors include the cytoplasmic tails of HA, NA (Enami & Enami, 1996; Jin *et al.*, 1997) and M2 (Iwatsuki-Horimoto *et al.*, 2006; McCown & Pekosz, 2006). Cellular candidates include membranes, as well as a number of M1-interacting proteins of possible significance to viral replication (Reinhardt & Wolff, 2000; Watanabe *et al.*, 2006).

The data presented here further demonstrate the multifunctional role of the middle domain of M1. In addition to its involvement in NP–RNP and –M1 interactions, previous studies have shown that the ^101^RKLKR^105^ sequence located in this domain mediates binding to RNA (Elster *et al.*, 1997), acts as a nuclear-localization signal (Ye *et al.*, 1995), interacts with nucleosomes (Garcia-Robles *et al.*, 2005), recruits NEP to enable RNP nuclear export (Akarsu *et al.*, 2003) and is involved in virus assembly (Burleigh *et al.*, 2005). Coordination of these different and possibly competing functions during the influenza A virus life cycle is likely to be partly regulated by M1's late temporal expression and its differential localization, in both the nucleus and the cytoplasm (Bucher *et al.*, 1989). Currently, the stoichiometry of the NEP–M1–RNP interaction necessary for nuclear export is unknown; however, it is likely to be low (Rey & Nayak, 1992; Whittaker *et al.*, 1995; Elton *et al.*, 2001, 2005). The stoichiometry of the M1–RNP interaction in virions is also unknown, but recent work regarding possible interactions between RNPs during genome packaging (Fujii *et al.*, 2003; Noda *et al.*, 2006), coupled with imaging of virus particles suggesting only limited regions of contact between the matrix layer and RNPs (Harris *et al.*, 2006), raises the possibility that this too is far lower than 1 : 1. Thus, oligomerized M1 may be able to mediate more than one function simultaneously by forming a meshwork in which individual monomers have non-equivalent functions. Indeed, such a suggestion has already been proposed to account for the ability of M1–NEP complexes to co-sediment with histones, even though both NEP and histones bind to the same region of the middle domain (Garcia-Robles *et al.*, 2005). Consistent with this model, we found that excess NP does not compete with WT M1 for binding to the middle domain of M1 (Fig. 6). However, the relationship between heterodimerization of NP and M1 and homopolymeric self-association is likely to be complex, with potentially negative effects resulting from competition for overlapping binding sites and positive effects resulting from polymeric increases in avidity. We suspect that these factors underlie the fact that titration of M1 sub-fragments (which can self-associate, but perhaps not polymerize) leads to a plateau in NP-binding activity, whilst higher amounts of the full-length M1 fusion protein display a lower binding capacity (Fig. 2b[Fig f2]). Further competition studies elucidating the hierarchy of M1 interactions may reveal how M1 mediates its multiple roles.
